# The economic burden of inpatient care of depression in Poznan (Poland) and Kiel (Germany) in 2016

**DOI:** 10.1371/journal.pone.0198890

**Published:** 2018-06-14

**Authors:** Tomasz Zaprutko, Robert Göder, Krzysztof Kus, Wiktor Pałys, Filip Rybakowski, Elżbieta Nowakowska

**Affiliations:** 1 Department of Pharmacoeconomics and Social Pharmacy, Poznan University of Medical Sciences, Poznan, Poland; 2 Department of Psychiatry and Psychotherapy, Christian-Albrechts-Universitat zu Kiel, Kiel, Germany; 3 Department of Adult Psychiatry, Karol Jonscher Clinical Hospital, Poznan University of Medical Sciences, Poznan, Poland; Jagiellonian University, POLAND

## Abstract

Depression is a global health problem associated with a significant public health burden and costs. Although studies on costs of diseases are being considered as an increasingly important factor for health policies, information concerning costs of inpatient care of depression is still insufficient. Thus, the main aim of this study was to evaluate costs of hospitalization of patients treated in 2016 in psychiatric clinics in Poznan (Poland) and in Kiel (Germany) and to analyze treatment used in these centers. The study was conducted from September 2017 to February 2018. 545 hospital records were considered (187 in Poznan and 358 in Kiel). Eventually, 490 hospital records were included, 168 in Poland and 322 in Germany. In general, the costs were calculated based on the patients’ sex and diagnosis (F32 and F33) separately and, subsequently, the outcomes were added and multiplied by the length of hospital stay, giving the cost of hospitalization. The annual cost of inpatient care of depression in 2016 was EUR 491,067.19 ($\bar{x}=\text{EUR}\;2923.02$) in Poznan and EUR 2,847,991.00 $\bar{x}=\text{EUR}\;8844.69$ in Kiel. In Poznan, hospitalization was underfunded reaching EUR 183,042.55 (37.27% of total costs in Poznan). In Poznan, the most frequently prescribed medicine was quetiapine, followed by olanzapine and venlafaxine, whereas in Kiel it was venlafaxine, followed by mirtazapine and promethazine. Although non-pharmacological therapies were commonly used in both centers, in Kiel this type of treatment was better structured. The study confirms the degree of the economic burden of inpatient care of depression. The underfunding of mental health revealed, emphasizes the need for urgent amendment of organization and funding of mental health care in Poland. Patients in Poznan were hospitalized on average 10 days longer than in Kiel, thus a reduction of length of hospitalization in Poznan seems possible. Although pharmacotherapy seemed to be comprehensive in both centers, there were some differences between Poznan and Kiel. Access to non-pharmacological therapies during outpatient care was limited in Poznan, however, compared to Kiel.

## Introduction

Depression is one of the most common, highly prevalent, and burdensome disorders worldwide [1–3], affecting people of any age [4]. According to the World Health Organization (WHO) data, depression is expected to become the second leading cause of disability or early death by 2020 [5]. The disease is characterized by a pertinaciously low mood with loss of interest in everyday activities [4], and might be associated with personal and public stigma [6]. It contributes to exacerbation of comorbid conditions such as hypertension or diabetes, for instance [7]. Moreover, untreated depression increases the risk of self-harm and suicide, and approximately two-thirds of suicide completers or attempters have major depressive episodes at the time of the suicidal act [7, 8].

Nevertheless, depression not only causes personal suffering but also produces significant economic burden both for the patients and the whole society being a major worldwide public health problem [9, 10]. Total costs of the disorder can be broken down into direct (e.g. inpatient and outpatient care, pharmacotherapy) and indirect costs (mainly related to the productivity loss) [9, 11]. In Europe, the total annual costs of depression were estimated at EUR 118 billion in 2004, with EUR 42 billion and 76 billion in direct and indirect costs, respectively [12]. These values were confirmed in a later study conducted by Olesen et al. [13], who indicated that major depression was among the most expensive disorders in relation to the total costs of brain disorders in 2010. In the United States, however, the economic burden of depression was USD 83 billion, with USD 26.1 billion allocated in direct costs and USD 56.9 billion in indirect costs [1]. Considering results from Asia likewise, Okumura and Higuchi [10] evaluated economic burden of depression in Japan with total costs estimated at USD 11 billion, with USD 4.1 billion in direct costs and USD 6.9 billion in indirect costs.

Although indirect costs represent the greatest share of the total costs of depression, direct costs are the significant part of the economic burden, too, with costs of inpatient care indicated as a significant [10] or even the most important contribution of direct costs generated by patients with depression [14]. In spite of this fact, there is still insufficient information concerning the costs of hospitalization of depression [10, 12, 15]. Hence, the main aim of this study was to investigate the costs of inpatient care of depression among patients hospitalized in 2016 in Poznan (Poland) and in Kiel (Germany). Moreover, the study was also to compare pharmacotherapy and non-pharmacological interventions used and to provide information about funding and organization of mental health care in the study centers. Poznan, with a population of approximately 550,000, is the capital of Greater Poland Voivodeship—Poland’s second largest province. Kiel, on the other hand, inhabited by a population of almost 250,000, is the capital city of the Schleswig-Holstein land, the northernmost state of Germany

## Material and methods

The study was conducted from September 2017 to February 2018. It evaluated costs of inpatient care of depression in 2016, at the Department of Adult Psychiatry of the Karol Jonscher Hospital of Poznan University of Medical Sciences (Poland) and at the Department of Psychiatry and Psychotherapy of the University Hospital Schleswig-Holstein of Christian-Albrechts University of Kiel (Germany).

Data were obtained from hospital records and from the hospital accounting departments. Before the analysis all data were fully anonymized, thus the study conforms with the Act on Protection of Personal Data. The costs were calculated based on the patients’ sex and diagnosis separately and, subsequently, the outcomes were added and multiplied by the length of hospital stay (LOHS), giving the cost of hospitalization. The structure of hospital records in Poznan allowed calculation of the costs of pharmacotherapy and diagnostic tests individually, and the results were presented as a percentage share of total costs.

In Kiel, however, all individual components (including hospital stay, pharmacotherapy, and diagnostic tests) up to the daily value of the procedure are covered, thus the cost of hospitalization in Kiel was not the direct result of multiplying LOHS and base rate of tariff per day, which was established by the health care payer. To evaluate the cost of inpatient care, each hospitalization was calculated separately in cooperation with the financial department of the hospital in Kiel. What is important, in Kiel the evaluation of the percentage contribution of e.g. pharmacotherapy would not have been possible because the hospital records present pharmacotherapy using international names or brand names marked frequently “for example” alternately, allowing the use of originators or any generic brands.

To analyze and compare pharmacotherapy, each medicine used was registered. Such detailed and meticulous analysis identified medications most frequently used in the study centers. For non-pharmacological interventions, however, hospital records, as well as freely available schedule of these therapies, were analyzed to compare the scope and types of non-pharmacological treatment in Poznan and Kiel.

Inclusion criteria were as follows: a diagnosed depression (in the study, two diagnosis codes were applicable: depressive episode—F32 and recurrent depressive disorder—F33) based on the International Classification of Diseases, Tenth Revision (ICD-10), and adult age of the patients (>18 years old). Patients were excluded from the study, however, if their LOHS was ≤ 3 days (this criterion was established because there were patients admitted to the hospital on Friday evening and discharged or transferred to another department on Monday morning) and if they left the hospital against medical advice.

In the study, 545 hospital records (all patients hospitalized in 2016 in Poznan and in Kiel) were taken into consideration (187 in Poznan and 358 in Kiel). However, based on the inclusion and exclusion criteria of the study, 490 hospital records were eventually included, n = 168 in Poland (106 women—W and 62 men—M; 70 diagnosed with F32 and 98 diagnosed with F33) and n = 322 in Germany (191 W and 131 M; 121 diagnosed with F32 and 201 diagnosed with F33). Results are presented as total costs of inpatient care of depression in Poznan and in Kiel and as average values associated with sex and diagnosis.

Due to the different currencies in Poland (PLN) and Germany (EUR), money values were converted from PLN to EUR at the average EUR exchange rate in 2016 published by the National Bank of Poland (EUR 1 = PLN 4.3625). Monetary values presented in the study are roundings of calculated amounts resulting from the conversion of monetary units into the common European currency. Moreover, results presented in EUR should make this paper clear and useful for the readers.

Furthermore, the study was approved by the Bioethics Committee of Poznan University of Medical Sciences and the Ethics Committee of Christian-Albrechts University in Kiel, as well as by hospital decision-makers in both Poznan and Kiel.

### Statistics

The data are shown as x ± SEM (plus the median and lower/upper quartile). Data distribution pattern was normal (like the Gaussian function). Statistically significant results (p<0.05) were demonstrated for homogenous groups using 2-ways Anova test and post-hoc Tukey test.

## Results

The mean age of patients in Poznan was 52.84 years. In Kiel, patients were a bit younger than in Poznan and the average age was 50.77. In terms of length of hospital stay, patients in Poznan were hospitalized on average more than 10 days longer than in the German hospital. Nevertheless, the shortest hospitalization included in both centers was 4 days regardless of the sex and diagnosis. On the other hand, the longest hospitalization in Poznan lasted 173 days (M; F33) and in Kiel 298 days (W; F33). In both centers, there were more W (106 in Poznan and 191 in Kiel) than M hospitalized (62 in Poznan and 131 in Kiel).

The annual cost of inpatient care of depression in 2016 was EUR 491,067.19 ($\bar{x}=\text{EUR}\;2923.02$) in Poznan and EUR 2,847,991.00 ($\bar{x}=\text{EUR}\;8844.69$) in Kiel. The cost for W was EUR 312,300.62 ($\bar{x}=\text{EUR}\;2946,23$) and EUR 1,782,064.00 ($\bar{x}=\text{EUR}\;9330.18$) in Poznan and Kiel, respectively. For M, it was EUR 178,766.57 ($\bar{x}=\text{EUR}$) in Poznan and EUR 1,065,927.00 ($\bar{x}=\text{EUR}\;8136.85$) in Kiel. The results of that task are presented in Tables 1, 2 and 3.

**Table 1:** Structure of the study group (Poznan).

**F32**			
	Total $\overset{-}{x}\pm SEM$	Women (Wo) $\overset{-}{x}\pm SEM$	Men (Me) $\overset{-}{x}\pm SEM$
Number of subjects	70	43	27
Mean age in years	48.91 ± 2.17 (M: 51.5 ~ L/U Q:33/65) NS (p = 0.5651) vs W NS (p = 0.4263) vs M	50.95 ± 2.81 (M: 53 ~ L/U Q:36/67)	45.67 ± 3.36 (M: 48 ~ L/U Q:28/59) NS (p = 0.2375) vs. W
Average duration of hospitalization in days	44.37 ± 2.50 (M: 49 ~ L/U Q:29/64) NS (p = 0.9178) vs W NS (p = 0.8892) vs M	44.79 ± 3.18 (M: 42 ~ L/U Q:28/66)	43.70 ± 4.13 (M: 49 ~ L/U Q:31/58) NS (p = 0.8345) vs. W
Cost of inpatient care	2730.62 ± 159.54 (M: 2817.81~ L/U Q:1729.75/3846.91) NS (p = 0.7608) vs W NS (p = 0.6840) vs M	2652.21 ± 199.83 (M: 2343.53~ L/U Q:1562.35/3846.91)	2855.48 ± 267.27 (M: 3084.45~ L/U Q:2168.26/4028.66) NS (p = 0.5391) vs W
Cost of pharmacotherapy	38.22 ± 5.94 (M: 21.46 ~ L/U Q:9.47/52.38) NS (p = 0.4789) vs W NS (p = 0.2523) vs M	45.56 ± 8.95 (M: 21.49 ~ L/U Q:10.89/57.68)	26.54 ± 5.32 (M: 15.65 ~ L/U Q:5.85/39.54) NS (p = 0.1200) vs. W
Cost of diagnostic tests	66.71 ± 3.97 (M: 57.54 ~ L/U Q:46.99/79.54) NS (p = 0.6078) vs W NS (p = 0.4910) vs M	70.00 ± 4.98 (M: 64.41 ~ L/U Q:46.99/85.73)	61.48 ± 6.57 (M: 49.05 ~ L/U Q:41.95/66.93) NS (p = 0.2989) vs W
Person-days used	3106	1926	1180
**F33**			
Number of subjects	98	63	35
Mean age in years	55.64 ± 1.58 (M: 58 ~ L/U Q:47/64) NS (p = 0.7201) vs W NS (p = 0.5792) vs M	54.71 ± 2.08 (M: 57 ~ L/U Q:47/66)	57.31 ± 2.37 (M: 59 ~ L/U Q:51/61) NS (p = 0.4343) vs. W
Average duration of hospitalization in days	49.13 ± 3.07 (M: 48.5 ~ L/U Q:24/68) NS (p = 0.5869) vs W NS (p = 0.4517) vs M	51.73 ± 3.54 (M: 52.0 ~ L/U Q:27/69)	44.46 ± 5.78 (M: 33.0 ~ L/U Q:21/63) NS (p = 0.2591) vs. W
Cost of inpatient care	3060.45 ± 189.76 (M: 2901.51 ~ L/U Q:1538.76/4266.57) NS (p = 0.7680) vs W NS (p = 0.6876) vs M	3146.91 ± 214.40 (M: 3287.36 ~ L/U Q:1841.34/4406.46)	2904.21 ± 369.31 (M: 2077.28 ~ L/U Q:1447.80/4126.69) NS (p = 0.5438) vs. W
Cost of pharmacotherapy	52.84 ± 8.52 (M: 30.56 ~ L/U Q:13.65/55.74) NS (p = 0.8690) vs W NS (p = 0.8115) vs M	55.07 ± 10.40 (M: 39.20 ~ L/U Q:19.83/64.32)	48.82 ± 14.95 (M: 13.65 ~ L/U Q:6.64/50.30) NS (p = 0.7271) vs. W
Cost of diagnostic tests	78.07 ± 4.69 (M: 66.71 ~ L/U Q:45.16/101.09) NS (p = 0.9296) vs W NS (p = 0.9060) vs M	77.44 ± 5.16 (M: 67.39 ~ L/U Q:48.37/93.75)	79.21 ± 9.41 (M: 57.54 ~ L/U Q:43.10/127.68) NS (p = 0.8573) vs. W
Person-days used	4815	3259	1556

M—median, L/U Q—lower and upper quartile, SEM—standard error of the mean, NS—statistically non-significant

**Table 2:** Structure of the study group (Kiel).

**F32**			
	Total $\overset{-}{x}\pm SEM$	Women (Wo) $\overset{-}{x}\pm SEM$	Men (Me) $\overset{-}{x}\pm SEM$
Number of subjects	121	70	51
Mean age in years	48.48 ± 1.85 (M: 45 ~ L/U Q:30/67) NS (p = 0.6527) vs Wo NS (p = 0.5524) vs Me	49.90 ± 2.64 (M: 48 ~ L/U Q:29/71)	46.53 ± 2.49 (M: 44 ~ L/U Q:32/56) NS (p = 0.3704) vs. Wo
Average duration of hospitalization in days	31.17 ± 2.10 (M: 28 ~ L/U Q:13/41) NS (p = 0.4307) vs Wo NS (p = 0.2779) vs Me	34.04 ± 3.12 (M: 28 ~ L/U Q:15/41)	27.22 ± 2.50 (M: 25 ~ L/U Q:13/38) NS (p = 0.1095) vs. Wo
Cost of inpatient care	7472.91 ± 489.09 (M: 6549 ~ L/U Q:3601/9601) NS (p = 0.4199) vs Wo NS (p = 0.2615) vs Me	8160.39 ± 732.10 (M: 6840.50 ~ L/U Q:3713/10075)	6529.31 ± 562.23 (M: 5944 ~ L/U Q:3214/9067) NS (p = 0.0998) vs. Wo
Person-days used	3771	2383	1388
**F33**			
Number of subjects	201	121	80
Mean age in years	55.15 ± 1.30 (M: 52 ~ L/U Q:38/67) NS (p = 0.6807) vs Wo NS (p = 0.5810) vs Me	53.03 ± 1.71 (M: 53 ~ L/U Q:38/71)	50.83 ± 1.98 (M: 51.5 ~ L/U Q:34.5/64) NS (p = 0.4058) vs. Wo
Average duration of hospitalization in days	40.33 ± 2.59 (M: 29 ~ L/U Q:15/55) NS (p = 0.7666) vs Wo NS (p = 0.6778) vs Me	41.62 ± 3.59 (M: 29 ~ L/U Q:14/58)	38.38 ± 3.61 (M: 30 ~ L/U Q:15/47) NS (p = 0.5412) vs. Wo
Cost of inpatient care	9670.49 ± 612.24 (M: 7086 ~ L/U Q:3713/13259) NS (p = 0.6491) vs Wo NS (p = 0.7423) vs Me	9061.65 ± 876.01 (M: 7069 ~ L/U Q:3758.5/10830)	10006.92 ± 837.49 (M: 7304 ~ L/U Q:3601/13716) NS (p = 0.5005) vs. Wo
Person-days used	8106	5036	3070

M—median, L/U Q—lower and upper quartile, SEM—standard error of the mean, NS—statistically non-significant

**Table 3:** Comparison between Poznan (Poland) and Kiel (Germany).

		Total $\overset{-}{x}\pm SEM$	Women (Wo) $\overset{-}{x}\pm SEM$	Men (Me) $\overset{-}{x}\pm SEM$	F 32 $\overset{-}{x}\pm SEM$	F 33 $\overset{-}{x}\pm SEM$
Number of subjects	PL D	168	106	62	70	98
		322	191	131	121	201
Mean age in years	PL D	52.84 ± 1.31 (M: 55 ~ L/U Q:42.5/64.5)	53.19 ± 1.69 (M: 55 ~ L/U Q:43/67)	52.24 ± 2.10 (M: 55.5 ~ L/U Q:39/61)	48.91 ± 2.16 (M: 51.5 ~ L/U Q:33/65)	55.64 ± 1.58 (M: 58 ~ L/U Q:47/64)
		50.77 ± 1.07 (M: 50 ~ L/U Q:34/67) NS (p = 0.2406)	51.89 ± 1.45 (M: 52 ~ L/U Q:34/71) NS (p = 0.5743)	49.15 ± 1.56 (M: 49 ~ L/U Q:34/60) NS (p = 0.2511)	48.48 ± 1.85 (M: 45 ~ L/U Q:30/67) NS (p = 0.8825)	52.15 ± 1.30 (M: 52 ~ L/U Q:38/67) NS (p = 0.1076)
Average duration of hospitalization in days	PL D	47.15 ± 2.08 (M: 49 ~ L/U Q:26.0/66.5)	48.92 ± 2.48 (M: 50 ~ L/U Q:28/69)	44.13 ± 3.70 (M: 43.5 ~ L/U Q:23/62)	44.37 ± 2.50 (M: 49 ~ L/U Q:29/64)	49.13 ± 3.07 (M: 48.5 ~ L/U Q:24/68)
		36.89 ± 1.81^x^ (M: 28 ~ L/U Q:14/50) **SS (p = 0.0005)**	38.84 ± 2.55^x^ (M: 28 ~ L/U Q:14/52) **SS (p = 0.0010)**	34.03 ± 2.45(M: 28 ~ L/U Q:14/44) **SS (p = 0.0221)**	31.17 ± 2.10^x^ (M: 28 ~ L/U Q:13/41) **SS (p = 0.0001)**	40.33 ± 2.29^x^ (M: 29 ~ L/U Q:15/55) **SS (p = 0.0409)**
Cost of inpatient care (EURO)	PL D	2923.02 ± 129.39 (M: 2870.66 ~ L/U Q:1657.65/3963.70)	2946.23 ± 152.23 (M: 2873.61 ~ L/U Q:1729.75/3961.68)	2883.33 ± 236.54 (M: 2811.71 ~ L/U Q:1468.82/4056.74)	2730.62 ± 159.54 (M: 2817.81 ~ L/U Q:1729.75/3846.91)	3060.45 ± 186.76 (M: 2901.51 ~ L/U Q:1538.76/4266.57)
		8844.69 ± 427.69^x^ (M: 6743.50 ~ L/U Q:3601/11658) **SS (p < 0.0001)**	9330.18 ± 596.77^x^ (M: 7052 ~ L/U Q:3601/13053) **SS (p < 0.0001)**	8136.85 ± 587.19^x^ (M: 6629 ~ L/U Q:3601/10287) **SS (p < 0.0001)**	7472.91 ± 489.09^x^ (M: 6549 ~ L/U Q:3601/9601) **SS (p = p < 0001)**	9670.49 ± 612.24^x^ (M: 7086 ~ L/U Q:3713/13259) **SS (p = p < 0001)**

L/U Q—lower and upper quartile, M—median, SEM—standard error of the mean, NS—statistically non-significant, SS—statistically significant

^x^ Statistically significant difference: D versus PL for p <0.05

In terms of diagnosis-related costs of inpatient care, the results were as follows. In Poznan, the cost of F32 was EUR 191,143.02 ($\bar{x}=\text{EUR}\;2730.62$) and of F33 –EUR 299,924.17 ($\bar{x}=\text{EUR}\;3060.45$). In Kiel, meanwhile, it was EUR 904,222.00 ($\bar{x}=\text{EUR}\;7472.91$) and EUR 1,943,769.00 ($\bar{x}=\text{EUR}\;9670.49$) respectively. The results of that task are presented in Tables 4 and 5.

**Table 4:** Results related to diagnosis (F 32/F 33)–Poznan.

	Total $\overset{-}{x}\pm SEM$	F 32 $\overset{-}{x}\pm SEM$	F 33 $\overset{-}{x}\pm SEM$
Number of subjects	168	70	98
Mean age in years	52.84 ± 1.31 (M: 55 ~ L/U Q:42.5/64.5) NS (p = 0.1130) vs F 32 NS (p = 0.1834) vs F 33	48.91 ± 2.17 (M: 51.5 ~ L/U Q:33/65)	55.64 ± 1.58^x^ (M: 58 **~** L/U Q:47/64) **SS (p = 0.0111) vs. F 32**
Average duration of hospitalization in days	47.15 ± 2.08 (M: 49.0 ~ L/U Q:26.0/66.5) NS (p = 0.4416) vs F 32 NS (p = 0.5813) vs F 33	44.37 ± 2.50 (M: 49.0 ~ L/U Q:29.0/64.0)	49.13 ± 3.07 (M: 48.5 ~ L/U Q:24.0/68.0) NS (p = 0.2597) vs. F 32
Cost of inpatient care	2923.02± 129.39 (M: 2870.66 ~ L/U Q:1657.65/3963.70) NS (p = 0.3943) vs F 32 NS (p = 0.5381) vs F 33	2730.62 ± 159.54 (M: 2817.81 ~ L/U Q:1729.75/3846.91)	3060.45 ± 189.76 (M: 2901.51 ~ L/U Q:1538.76/4266.57) NS (p = 0.2098) vs. F 32
Cost of pharmacotherapy	46.75 ± 5.56 (M: 26.51 ~ L/U Q:10.65/54.97) NS (p = 0.3674) vs F 32 NS (p = 0.5334) vs F 33	38.22 ± 5.94 (M: 21.46 ~ L/U Q:9.47/52.38)	52.84 ± 8.52 (M: 30.56 ~ L/U Q:13.65/55.74) NS (p = 0.1962) vs. F 32
Cost of diagnostic tests	73.34 ± 3.22 (M: 63.50 ~ L/U Q:46.07/88.71) NS (p = 0.2386) vs F 32 NS (p = 0.3929) vs F 33	66.71 ± 3.97 (M: 57.54 ~ L/U Q:46.99/79.54)	78.07 ± 4.69 (M: 66.71 ~ L/U Q:45.16/101.09) NS (p = 0.0818) vs. F 32
Person-days used	7921	3106	4815

L/U Q—lower and upper quartile, M—median, SEM—standard error of the mean, NS—statistically non-significant, SS—statistically significant

^x^ Statistically significant difference: F 33 versus F 32 for p <0.05

**Table 5:** Results related to diagnosis (F 32/F 33)—Kiel.

	Total $\overset{-}{x}\pm SEM$	F 32 $\overset{-}{x}\pm SEM$	F 33 $\overset{-}{x}\pm SEM$
Number of subjects	322	121	201
Mean age in years	50.77 ± 1.07 (M: 50 ~ L/U Q:34/67) NS (p = 0.2709) vs F 32 NS (p = 0.4163) vs F 33	48.48 ± 1.85 (M: 45 ~ L/U Q:30/67)	52.15 ± 1.30 (M: 52 ~ L/U Q:38/67) NS (p = 0.0962) vs F 32
Average duration of hospitalization in days	36.89 ± 1.81 (M: 28 ~ L/U Q:14/50) NS (p = 0.7732) vs F 32 NS (p = 0.2636) vs F 33	31.17 ± 2.10 (M: 28 ~ L/U Q:13/41)	40.33 ± 2.59^x^ (M: 29 ~ L/U Q:15/55) **SS (p = 0.0142) vs F 32**
Cost of inpatient care	8844.69 ± 427.69 (M: 6743.5 ~ L/U Q:3601/11658) NS (p = 0.0716) vs F 32 NS (p = 0.2558) vs F 33	7472.91 ± 489.09 (M: 6549 ~ L/U Q:3601/9601)	9670.49 ± 612.24 (M: 7086 ~ L/U Q:3713/13259) **SS (p = 0.0126) vs F 32**
Person-days used	11877	3771	8106

M—median, L/U Q—lower and upper quartile, SEM—standard error of the mean, NS—statistically non-significant, SS—statistically significant

^x^ Statistically significant difference: F 33 versus F 32 for p <0.05

Although all components of the total cost (cost of hospital stay, pharmacotherapy and diagnostic tests) were included into the value of the daily medical procedure in both centers, the structure of hospital records in Poznan allowed a separate evaluation the cost of pharmacotherapy and diagnostic tests. The value of pharmacotherapy used was EUR 7,853.95, which corresponds to 1.60% of total costs in Poznan. The cost of medicines used generated by W was EUR 5,428.56 (F32 –EUR 1,959.00 and F33 –EUR 3,469.56) and by M—EUR 2,425.39 (F32 –EUR 716.61 and F33–1,708.78). Values per patient are depicted in Tables 1 and 4.

The cost of diagnostic tests was EUR 12,320.45, which corresponds to 2.51% of total costs of hospitalization in Poznan. The value generated by W was EUR 7,888.59 (F32 –EUR 3,009.97 and F33 –EUR 4,878.62) and by M—EUR 4,431.86 (F32 –EUR 1,659.37 and F33 –EUR 2,772.49). Values per patient are depicted in Tables 1 and 4.

In both centers, there was a tariff rate per day established by the healthcare payer. In Poznan, it was EUR 39.54 decreasing to EUR 27.68 per person per day for each day of hospitalization exceeding 70 days. Nevertheless, this value was insufficient from the hospital’s point of view, with the costs per day (including hospital stay, pharmacotherapy, and diagnostic tests) amounting to EUR 55.80 at the men’s ward, EUR 62.95 at the women’s ward, and EUR 69.94 at the mixed ward. The difference in the pricing of tariff rate per day by the hospital and the healthcare payer allowed us to evaluate the degree of underfunding of mental health care at the Polish hospital.

Detailed analysis of values of tariff rates per day found that inpatient care of depression was underfunded in Poznan by as much as EUR 183,042.55, which corresponds to 37.27% of total cost at the Polish hospital. This results from the daily value of procedure funded by the Polish healthcare payer which leads to the underfunding from the very first day of hospitalization and exacerbates the problem in case of hospitalizations lasting more than 70 days.

In Kiel, however, the base tariff rate per day was EUR 259.71 regardless of the patient’s sex and the ward of hospital stay. In spite of the fact that the value of daily rate differed between particular cases of hospitalization, the final cost of each hospitalization was indicated as sufficient to cover all expenses related to inpatient care; thus, in Germany there was no underfunding problem.

Apart from the economic burden, it might be interesting to analyze treatment schedules used in Poznan and in Kiel. Although treatment in both centers could be defined as comprehensive, there were some differences in this respect. In terms of pharmacotherapy used, the most frequently used substances in Poznan were quetiapine (used by 53.57% of patients), olanzapine (used by 32.74% of patients), and venlafaxine (used by 26.79% of patients). These substances were followed by hydroxyzine and haloperidol, prescribed to 23.81% and 19.64% of patients, respectively. In Kiel, however, venlafaxine was the most popular substance used by 30.43% of patients, followed by mirtazapine and promethazine prescribed to 28.88% and 22.36% of patients, respectively. Citalopram was the fourth most frequently used drug, at 16.15%, followed by olanzapine prescribed to 15.22% of hospitalized patients. Interestingly, only 1 patient in Poznan received promethazine. On the other hand, no one in Kiel was treated with hydroxyzine or haloperidol. Both in Poznan (73.81%) and in Kiel (54.04%), most patients were treated with pharmacotherapy related to concomitant disorders such as diabetes or hypertension for instance. Considering benzodiazepines in general, this group of medicines was prescribed to 55.36% of patients in Poznan and to 18.32% of those hospitalized in Kiel.

In both centers, pharmacotherapy was supported with non-pharmacological interventions. Nevertheless, it seemed to be more extensive in Kiel mainly due to the wide offer of trainings which were freely available also in outpatient care. Apart from psychoeducation, music therapy, and ergotherapy, other popular options in Kiel included light therapy (LT), gymnastics, Nordic Walking, or bathing in cold water known as “kneippen” in Germany. Patients in Kiel had 6 or 7 daily options of various non-pharmacological interventions which were individually fixed. In Poznan, however, the offer of non-pharmacological therapies used was slightly limited in comparison to Kiel. There was no “kneippen” or LT, for instance. Nevertheless, psychoeducation, occupational therapy, gymnastics, and others were popular, too. The number of daily training options was smaller than in Kiel, however. Moreover, the problem of non-pharmacological therapy in Poznan seems to be related to the lack of proper facilities in outpatient care where patients would be able to continue therapies started during hospitalization.

## Discussion

Depression is considered one of the disorders characterized by the greatest costs and burden for the society as well as for the public healthcare system [1, 9, 16]. In terms of economic burden, depression is primarily related to indirect costs, but direct costs are responsible for a significant part of that burden, too [9, 10, 17, 18]. In addition to this, a study conducted by Kleine-Budde et al. [9] identified costs of hospitalization as the main component of direct costs of depression. In spite of this fact, there is still insufficient information concerning costs on inpatient care of depression [9, 10, 12, 15], thus the importance of this study. The evaluation of total cost of hospitalization in 2016, amounting to EUR 491,067.19 ($\bar{x}=\text{EUR}\;2923.02$) in Poznan (n = 168) and EUR 2,847,991.00 ($\bar{x}=\text{EUR}\;8844.69$) in Kiel (n = 322), confirms the significance of costs related to inpatient care and corroborates with other studies where authors emphasized the degree of economic burden of depression [9, 10, 15, 19].

Although the general trend in depression costs analysis is convergent between individual studies and confirms the economic burden of that disorder, differences between results from various countries might be meaningful. For example, Okumura and Higuchi compared annual direct medical costs of depression in Japan (USD 689 per patient) against outcomes from Spain (USD 1166 per patient) and the USA (USD 1400 per patient) [10]. Additionally, in the systematic review of the cost of illness studies, mean direct costs per patient ranged from USD 1000 to USD 2500 annually [20]. Considering these variances and the fact that an average hospitalization lasted 10 days longer in Poznan (47 days) than in Kiel (37 days), the 3-fold discrepancy between average costs of hospitalization in the study centers seems hardly surprising. Moreover, it could be even greater if hospitalizations had been equalized in terms of LOHS. Discrepancies in LOHS for patients with depression are quite common and range from 61 days in Canada, through 51 days in Germany, to 11 days in the USA [21, 22].

Nonetheless, as in our study, costs differences might be the effect of dissimilarities in funding of healthcare systems, too. Furthermore, these considerable variances in costs analysis also depend on settings and methodology used in the studies and might be affected by differences in economic factors and pharmaceutical costs among countries likewise [10, 19, 20, 23]. Considering, for instance, economic facets it is important to point out that the German economy is the fifth largest economy in the world in Purchasing Power Parity (PPP) terms and Europe’s largest, whereas Poland has the sixth-largest economy in the European Union [24]. According to data from 2016 Gross Domestic Product (GDP) per capita and PPP accounted for USD 28.200 and 1.788 (National currency units/US dollar) in Poland respectively. In Germany, however, it was USD 49.300 and 0.780 (National currency units/US dollar) accordingly [24, 25]. These data confirm the impact of the economy on hospitalization costs differences observed between Poznan and Kiel, hence the importance of many factors which are components of economic and public health burden of disease.

Many studies indicate that more women than men are diagnosed with depression [6, 26] and these findings corroborate with the results of our study. In general, men are known to reveal a reluctance to present concerns about their mental health e.g. due to socioculturally prescribed male roles related to gender-relevant behavior [21, 27], and they do not seek mental health care as often as women do [6, 28]. Moreover, typical symptoms of depression might be masked among men by other signs related to men’s tendency to be overly sexually active, usually in the form of promiscuity or a series of brief affairs [27]. Nevertheless, as revealed in our study, men in both Poznan and Kiel were hospitalized shorter than women and this finding is in line with results obtained in Japan [21]. On the one hand, it could be deemed surprising, especially considering the fact that hidden symptoms of depression among men may contribute to a more severe course of depression. On the other hand, however, it could be related to potential gender differences in the response to pharmacotherapy used and co-existing eating disorders as well as anxiety which are more likely to affect women [29]. Nonetheless, this emphasizes the need to carefully investigate potential facets related to gender differences affecting the economic burden of depression.

Considering pharmacological treatment of depression, selective serotonin reuptake inhibitors (SSRI e.g. citalopram) and serotonin-norepinephrine reuptake inhibitors (SNRI e.g. venlafaxine) as well as noradrenergic and specific serotonergic antidepressants (NaSSA e.g. mirtazapine), are recommended, for instance, by the American Psychiatric Association as the first-line treatment of depression [4, 14, 30]. Nevertheless, because of the effect on the multiple receptor systems, concurrent use of antidepressants and antipsychotics (both typical and atypical) is considered more effective than monotherapy with antidepressants [31]. Therefore, the use of antipsychotics has been one of the most important strategies aimed at a more effective treatment of depression [31] and within the last decade aripiprazole, quetiapine, and olanzapine were approved by the US Food and Drug Administration (FDA) as an augmentation to antidepressant therapy in depression [30]. Results of the analysis of medicines used in Poznan and Kiel corroborate with these findings and confirm that the pharmacotherapy applied was comprehensive and up-to-date in both centers. There were some differences between Poznan and Kiel, however. In the Polish hospital, antipsychotics (quetiapine and olanzapine) were the most frequently used, followed by venlafaxine (SNRI). In Kiel, however, it was venlafaxine, followed by mirtazapine (NaSSA), and promethazine (H1-receptor antagonist; responsible for e.g. sedative effect). Although antipsychotics were also frequently used in Kiel, popularity of venlafaxine and mirtazapine might result not only from the patients’ health needs but also from local conditions because these substances were identified by Warnke et al. [14] as more preferred in Germany than in other European countries. It is worth noting that both hospitals used benzodiazepines frequently as well. This is in line with the study conducted in Brazil by Cigognini et al. where benzodiazepines were the most frequently prescribed medicines, followed by fluoxetine [32]. The above-mentioned study, however was carried out in 2002 and fluoxetine, for instance, is not so popular right now, in contrast to benzodiazepines which are still used frequently. Furthermore, majority of the patients in Poznan and in Kiel were treated for comorbid conditions. Hypertension, coronary heart disease, diabetes, or hyperlipidemia were commonly observed in other studies likewise [4]. These frequently chronic disorders may significantly affect the economic burden of inpatient care of depression, mainly due to the possible impact on LOHS.

In spite of the fact that we were unable to calculate the percentage share of pharmacotherapy in total cost of hospitalization because of the structure of hospital records and funding of mental health care in Kiel, hospital staff mentioned a growing share of generic drugs. Interestingly, based on the information obtained from the hospital staff, costs of pharmacotherapy decreased significantly and constituted approximately 2% of total costs of inpatient care, which is consistent with the results obtained in Poznan.

Apart from pharmacotherapy, non-pharmacological interventions are recommended as a concomitant treatment of depression as well [4]. Several non-pharmacological therapies like cognitive behavioral therapy, psychotherapy, or exercise therapy are considered effective also in terms of relapse rates [5, 33] and might, thus, contribute to a decrease of depression costs, especially in the long run. Comparing these interventions between Poznan and Kiel, non-pharmacological therapies at the Polish hospital seemed to be less structured. In Kiel, for instance, owing to the many daily training options, non-pharmacological treatment could be more customized. Aside from quite popular trainings, such as psychoeducation, patients hospitalized in Kiel attended “kneippen” or LT. Nonetheless, Farah et al. [33] claimed that several non-pharmacological interventions are characterized by similar effectiveness, hence non-pharmacological therapies of patients in Poznan could be considered as sufficient and equally effective as those in Kiel. On the other hand, especially LT seems to be quite easy and cheap to incorporate and, according to the study conducted by Winkler-Pjerk et al. [34], this method is not only frequently used in Germany, Austria and Switzerland, but is known as an effective intervention too.

These therapies should be also available, however, in outpatient care to provide a comprehensive and the most effective treatment. Nevertheless, access to non-pharmacological interventions for patients discharged from hospital seems to be limited in Poznan compared to Kiel, mainly due to the lack of suitable facilities. In Germany, there are also many employment possibilities after a mental health crisis, making the unemployment rate among the mentally ill significantly lower than in other countries [35, 36]. In addition to this, employment is therapeutic and reduces the risk of hospital readmissions among patients suffering from mental disorders [14, 36, 37]. Moreover, employment of those people could help reduce the social and self-stigma [6] as well as indirect (productivity loss) and direct costs (hospitalization rates) [9]. From this study’s perspective, savings in terms of direct medical costs are crucial. It is particularly important in Poznan where mental healthcare turned out to be significantly underfunded by the national healthcare payer. This statement is in line with the study carried out by Zaprutko et al. [38] where authors revealed the same problem in Poland and the Ukraine. In spite of the fact that the present study demonstrated that treatment applied at the Polish hospital was comprehensive and state-of-the-art, findings related to underfunding confirm the urgent need of improvement of mental healthcare funding in Poland. It also shows that the development of facilities providing daily care with additional non-pharmacological therapies could pay off in terms of public health and economic burden of depression, especially because mental well-being is crucial for achievement of the strategic objectives of the European Union health policy [39].

Our study has some limitations, though. It would be very interesting to study more hospitals from Poland and Germany and roll the study out to other countries afterwards. Considering insufficient number of studies related to economic burden of inpatient care of depression, however, this study might be recognized as an important contribution in the field. In spite of the fact that funding of mental healthcare in Germany and structure of hospital records prevented us from presenting the exact percentage share of pharmacotherapy and diagnostic tests in total cost of hospitalization, it would be interesting for the readers to learn such information. It could also be worthwhile to perform a prospective analysis of this issue, as it would help us collect the patients’ opinions about their feelings on various non-pharmacological interventions and the issue of public and personal stigma. Furthermore, the analysis of the marital status and family structure could provide interesting information about the possible effect on LOHS and, thus, costs of inpatient care of depression. Besides, it could be interesting to conduct a detailed analysis of ICD-10 codes (F32 from F32.0 to F32.3 and F33 from F33.0 to F33.3) of patients admitted to the hospital. Thus, if Kiel admits only severe patients and Poznan admits only moderate patients, or inversely, it could have the impact on presented results related to LOHS and costs of inpatient care likewise. Nonetheless, costs of depression are frequently presented under the general term “depression” or “major depressive disorder”. On the other hand, some authors [10, 40] decided to use diagnosis codes in their analyses but only F32 and F33 were applicable and, after a very careful consideration, we decided to follow them and analyze F32 and F33 separately. However, as indicated by the Heads (they are also co-authors of this study) of Psychiatry Departments in Poznan and in Kiel there were mixed diagnoses, hence presented results might be considered as valuable and comparable too. Moreover, the value of the study would be higher if we were able to present information about the number of staff employed in the study centers (physicians, nurses, non-pharmacological therapists). It would be also valuable to present more economic factors (e.g. Gini coefficient) and to implement International Dollar in the analysis, which is a hypothetical currency aimed at explaining and comparing prices from one country to another. Nevertheless, the authors decided to use the European currency to ensure clarity of the text also for readers who are not specialists in the field. Another limitation is related to a possibly interesting new point in terms of the economic burden of multiple disorders. Evaluation of the cost of translation could be interesting as well. In Kiel, in the case of some immigrants, there was a real language barrier, requiring a temporary employment of translators. Although it is difficult to compare costs in different countries, this study could be considered a valuable source of data on the economic burden and treatment of depression.

## Conclusions

Although this study confirms the significant economic burden of depression in terms of hospitalizations costs, it also emphasizes the need of urgent improvement of mental health care funding in Poland, especially due to the underfunding observed. Patients in Poznan were hospitalized on average 10 days longer than in Kiel which confirms that a reduction of LOHS in Poznan seems possible. In spite of the fact that pharmacotherapy was responsible for a low percentage share of total costs of inpatient care, treatment was comprehensive in both centers. Nevertheless, access to non-pharmacological therapies during outpatient care was limited in Poznan compared to Kiel.

## Supporting information

S1 Table Medicines used in Kiel. This is the list of medicines used in Kiel.
(XLSX)

S2 Table Costs in Poznan. This is the list of costs’ components of costs of inpatient care of depression in Poznan.
(XLSX)

S3 Table Medicines used in Poznan. This is the list of medicines used in Poznan.
(XLSX)

S4 Table Costs in Kiel. This is the list of costs’ components of costs of inpatient care of depression in Kiel.
(XLSX)
