# Correction: The economic burden of inpatient care of depression in Poznan (Poland) and Kiel (Germany) in 2016

**DOI:** 10.1371/journal.pone.0200684

**Published:** 2018-07-11

**Authors:** Tomasz Zaprutko, Robert Göder, Krzysztof Kus, Wiktor Pałys, Filip Rybakowski, Elżbieta Nowakowska

In the Funding section, the grant number from the funder National Science Centre, Poland is listed incorrectly. The correct grant number is: 2017/01/X/NZ7/00055.
